# Should We Prescribe More Protein to Critically Ill Patients?

**DOI:** 10.3390/nu10040462

**Published:** 2018-04-07

**Authors:** Daren K. Heyland, Renee Stapleton, Charlene Compher

**Affiliations:** 1 Department of Critical Care Medicine, Kingston General Hospital, Kingston, ON K7L 2V7, Canada; 2 Department of Public Health Sciences, Queen’s University, Kingston, ON K7L 3N6, Canada; 3 Clinical Evaluation Research Unit, Kingston General Hospital, Kingston, ON K7L 2V7, Canada; 4 Pulmonary and Critical Care Division, University of Vermont College of Medicine, Burlington, VT 05405, USA; Renee.Stapleton@uvm.edu; 5 Biobehavioral Research Laboratory, University of Pennsylvania, Philadelphia, PA 19104, USA; compherc@nursing.upenn.edu

**Keywords:** EFFORT trial, NEXIS trial, high protein, critical care nutrition, critically ill, protein supplementation

## Abstract

In the context of critical illness, evidence suggests that exogenous protein/amino acid supplementation has the potential to favorably impact whole-body protein balance. Whether this translates into retention of muscle, greater muscle strength, and improved survival and physical recovery of critically ill patients remains uncertain. The purpose of this brief commentary is to provide an overview of the clinical evidence for and against increasing protein doses and to introduce two new trials that will add considerably to our evolving understanding of protein requirements in the critically ill adult patient.

## 1. Introduction

In the context of critical illness, evidence suggests that exogenous protein/amino acid supplementation has the potential to favorably impact whole body protein balance [1,2]. Whether this translates into the retention of muscle, greater muscle strength, and improved survival and physical recovery of critically ill patients remains uncertain. The American Society for Parenteral and Enteral Nutrition (ASPEN) and the Society of Critical Care Medicine (SCCM) nutrition guidelines recommend 1.2 to 2.0 g/kg/day [3]. Some experts conclude that up to 2.0–2.5 g/kg/day of protein, and even higher doses in severe burn and trauma patients, is safe and could be considered an optimal dose [4]. Yet current observational studies document that critically ill patients are being prescribed much less than that, an average of 1.3 g/kg/day, and receiving only 55% of what is prescribed on average (approximately 0.7 g/kg/day) [5]. Is increasing protein delivery warranted? The purpose of this brief commentary is to provide an overview of the clinical evidence for and against increasing protein doses and to introduce two new trials that will add considerably to our evolving understanding of protein requirements in the critically ill adult patients.

## 2. What Does the Evidence Say?

**Figure 1:**
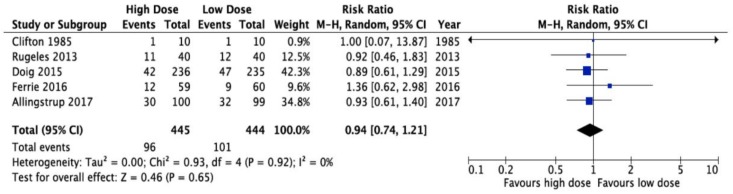
Meta-analysis of five randomized trials of high vs. low dose protein administration in the critically ill: effect on overall mortality.

There are only five Randomized Clinical Trials (RCTs) of Intensive Care Unit (ICU) patients specifically randomized to a high versus a lower protein intake, Figure 1 [6,7,8,9,10]. These trials vary in sample size (20–470), methodological quality (scores of 7–10 out of 14), year of publication (1985–2017), the patients studied, the protein doses prescribed, and the outcomes assessed (see Table 1). The trial by Clifton [9] (*n* = 20) was so small that they did not demonstrate any differences in clinical outcomes. The trial by Rugeles and The Early Goal-Directed Nutrition in ICU Patients (the EAT-ICU trial) also failed to show a difference in outcome, but the increased protein dose was confounded by increased energy, as well as the group that obtained more protein received more energy [8]. In the EAT-ICU trial, this results in significantly worse glycemic control, which may have negated any positive effect of more protein [10]. Moreover, in the EAT-ICU trial, investigators chose the physical component summary scale (PCS) as their primary outcome. This measure includes items that may not be responsive to nutritional interventions and, as evidenced by the wide confidence limits, the trial was underpowered to detect a treatment effect on PCS (adjusted mean difference 0.0, 95% confidence limits −5.9, 5.8). Ferrie and colleagues did demonstrate a significant improvement in muscle mass and a trend towards increased handgrip strength in the group that received higher protein but differences in protein received between the two groups were small (1.1 g/kg/day vs. 0.9 g/kg/day) [6]. Due to the heterogeneity of outcome assessment and incomplete data sets, we were only able to aggregate the effect of higher protein dosing on mortality (risk ratio 0.94, 95% CI 0.74–1.21, *p* = 0.65, see Figure 1), though, admittedly, none of these trials was adequately powered to assess mortality. Thus, the RCTs in the field, which are few and of varying quality and significance, do not settle the controversy about the optimal role of protein delivery.

Robust statistical analyses of large, multicenter observational databases show that for an additional 30 g of protein per day, or 1000 calories per day received during the first 12 days of ICU stay, critically ill patients have reduced infectious complications, shorter duration of mechanical ventilation, improved short-term physical recovery, and reduced mortality [11,12,13,14]. In another recent analysis [15], we demonstrated that meeting protein requirements seems to be more important than meeting caloric requirements. When we control for caloric intake, we still see a significant reduction in associated mortality when more than 80% of protein requirements are delivered compared to less than 80% (odds ratio (OR) for 60 days mortality 0.68, and 95% confidence interval (CI): 0.50, 0.91). In contrast, when we control for protein administration, there is no incremental effect of increased caloric administration (OR 0.89; 95% CI 0.71, 1.12). Whilst the inference is weak from this statistical modelling, it is consistent with other observational studies that show an association between protein optimization and survival, but a negative, or absent, effect of caloric intake [16,17].

In contrast to the prevailing data, some observational studies have reported that adverse patient outcomes were associated with higher protein intake. In an elegant cohort study that carefully examined skeletal muscle during critical illness where included patients were provided mostly enteral nutrition and received approximately 0.67 g/kg/day, Puthucheary and colleagues concluded that increased protein delivery during the first 10 days of ICU stay was associated with increased muscle wasting [18]. In a post-hoc analysis of the The Early Parenteral Nutrition Completing Enteral Nutrition in Adult Critically Ill Patients (EPaNIC)trial [19], investigators showed an association with increased protein intake during the first three days and lower likelihood of early ICU discharge [20]. Finally, others have published a post-hoc analysis of a small randomized trial of aggressive nutritional interventions where patients received approximately 80 g of protein per day compared to a usual care group which received around 60 g/day, and demonstrated that the amount of protein received in the first week was associated with a significant increased risk of death, whereas protein provided after the first week seemed protective [21]. Whilst these observations are hypothesis-generating analyses and have flaws that have been contested in the literature [22,23], they are significant in that they suggest a significant harm associated with increased protein, particularly in the acute phase of illness, and further contribute to the uncertainty about the role of protein in critical illness.

This conflict between observational and interventional studies can, in part, be resolved using our nascent understanding of nutrition risk assessment in the critically ill. Nutritionally high-risk means those patients populations that are more likely to respond to optimal nutrition intake and have better clinical outcomes than those with lower nutritional risk. Large-scale RCTs may have failed to demonstrate an impact of different amounts of nutrition intake because they enroll heterogeneous patient populations of varying nutritional risk, not all of whom will respond to optimal nutrition intake. The evidence for this assertion comes from observational studies that demonstrate a differential treatment effect of artificial nutrition in different subgroups of ICU patients [12]. The Nutrition Risk in Critically ill (NUTRIC) score aids in identifying the patient group that benefits the most from optimal nutrition and the score has been validated in several datasets representing diverse patient populations [24,25,26]. However, in a recent post-hoc analysis of the PERMIT (Permissive Underfeeding versus Target Enteral Feeding in Adult Critically Ill Patients) trial, this concept of a different treatment effect in patients with high vs. low nutrition risk [27]. However, this analysis was underpowered and focused on patients with different levels of calories, not protein. Another recent post hoc subgroup analysis did show a large survival advantage in the group of patients with a high NUTRIC score receiving supplemental PN compared to those patients with high NUTRIC scores receiving only enteral nutrition, whereas no signal of treatment effect of supplemental parenteral nutrition was observed in patients with low NUTRIC scores [28]. In nutritionally high-risk patients, evaluating the effect of a higher dose of protein administration, compared to usual low-dose protein administration, is a high priority research question [29].

## 3. Does the Combination of Protein and Exercise Hold Promise? 

The administration of higher doses of protein, compared to usual care where patients are receiving approximately 0.7 g/kg/day, may translate into improved outcomes of critically ill patients but, most likely, the treatment effect is greater under conditions of exercise. Prior rehabilitation trials have not reported nutrition intake, or have grossly underfed their patients [30,31,32,33]. Past trials in various non-critically ill populations, combining protein and exercise interventions have demonstrated the largest beneficial treatment effects in terms of preserving muscle mass, strength or function compared with either nutrition or exercise alone [34,35]. Exploring the effect of the combined effects of nutrition and exercise in critically ill patients compared to usual care represents the most important research priority in our clinical nutrition community [27].

## 4. Is It Safe to Administer Higher Doses of Protein/Amino Acids?

Experts who have recently extensively reviewed the literature to assess the safety of high dose protein/amino acid administration concluded that up to 2.5 g/kg/day are safe in general ICU patients except, perhaps, in patients with refractory hypotension (which causes hypoperfusion of the liver) and serious liver disease [3]. Patients with renal failure are a special population. Since patients in acute renal failure requiring renal replacement therapy lose such a greater amount of amino acids in the dialysate effluent, it is thought that they require higher doses of protein/amino acid administration, up to 2.5 g/kg/day. However, the strongest evidence for this assertion comes from one RCT of 50 patients randomized to two different groups that had similar nitrogen intake, showing that patients that received an escalating dose (compared to a fixed dose) had better nitrogen balance [35]. There is a rationale that intravenous amino acids may be helpful for renal function, supposedly by improving renal perfusion. Based on this concept, Doig et al. conducted a large RCT of IV (Intravenous) amino acids of up to 2.0 g/kg/day in 474 ICU patients compared to protein from standard enteral nutrition alone [7]. There was slight improvement in the estimated glomerular filtration rate, but no difference in dialysis rates or other clinical outcomes. Both fluid intake and urine output were increased significantly in the high protein group, but fluid balance was unchanged. It is important to realize that such a high dose of amino acids may increase ureagenesis and serum urea levels relative to what is seen in standard clinical practice (16 mmol/L (45 mg/dL) vs. 11 mmol/L (31 mg/dL) in the non-supplemented group). An isolated rise in serum urea is not known to be harmful and, by itself, is not an indication for earlier dialysis. The Doig RCT does not support the hypothesis that more protein is associated with better outcomes in a diverse patient population, though it was admittedly underpowered to detect such outcomes; however, we can point to this trial to demonstrate the safety of this approach, particularly in patients at risk of kidney injury.

## 5. More Information Needed!

Clearly definitive proof from prospective RCTs evaluating different levels of protein intake in nutritionally high-risk patients is lacking. Fortunately, upcoming trials will help resolve this controversy. Funded by the National Institutes of Health, the NEXIS Trial (Nutrition and EXercise in Critical Illness: A Randomized Trial of Combined Cycle Ergometry and Amino Acids in the ICU)will evaluate the effect of early bedside cycling, an innovative physical activity intervention, and intravenous amino acids (to a maximum of 2.5 g/kg/day) on the physical recovery of long-stay ICU patients. Early bedside cycling has been shown to be both feasible and safe in this patient population and may impact short-term functional outcomes [32,36]. The primary outcome of the NEXIS trial is the six minute walk test and a comprehensive set of outcomes that will characterize the treatment effect on muscle mass, muscle strength, and functional capacity, and quality of life will be conducted in accordance with a recent consensus statement [37]. This trial began enrollment in the summer of 2017 and results are expected sometime in 2021 [38].

At the same time, researchers are designing a large, multicenter, pragmatic, volunteer-driven, registry-based, randomized clinical trial of 4000 nutritionally high-risk critically ill patients who will be randomly allocated to a higher dose of protein (≥2.2 g/kg/day) or usual care (≤1.2 g/kg/day), known as the EFFORT (The Effect of Higher Protein Dosing in Critically Ill Patients) trial [39]. All other aspects of clinical care will be dictated by local standards, but participating sites will be encouraged to avoid overfeeding calories and to follow the ASPEN/SCCM guidelines regarding the amount of calories that should be prescribed. Perhaps what is unique to this large-scale RCT is that only ‘nutritionally high-risk patients’ will be enrolled. Nutrition risk is defined by one of the following: (1) low (≤25) or high BMI (≥35); (2) moderate to severe malnutrition (as defined by local assessments); (3) frailty (Clinical Frailty Scale of 5 or more from proxy); (4) sarcopenia- (SARC-F score of 4 or more from proxy); or (5) from the point of screening, projected duration of mechanical ventilation >4 days. Consideration was given on whether to include or exclude various subgroups of patients who might have higher protein requirements (renal failure, burns, trauma, or obesity, for example) or lower requirements (liver disease or older patients, for example), but since the evidence for dosing these subpopulations is uncertain and provider beliefs on what is best are variable, we reasoned to not exclude them and have planned several a priori subgroup analysis to evaluate the effect of protein administration in these important subgroups. The primary outcome for this trial is 60-day mortality and secondary outcomes include time-to-discharge-alive, nutritional adequacy, hospital mortality, readmission to ICU and hospital, and duration of mechanical ventilation, ICU stay, and hospital stay. The EFFORT trial begins enrollment in January 2018 (see www.criticalcarenutrition.com for more information). To participate in these trials, clinicians must have ‘equipoise’ and believe that either dosing strategy is safe and may be efficacious.

## 6. Conclusions

There is an insufficient body of literature to inform clinical practice guidelines as to the optimal dose of protein that should be prescribed to critically ill patients. Fortunately, new trials will shed light on the impact of protein administration in this setting. When available, the results of these trials will give greater confidence as to the optimal dose of protein for critically ill patients.

## Figures and Tables

**Table 1:** Randomized trials of high vs. low protein/amino acid doses.

Study	Population	Methods (Score)	Intervention	Mortality # (%) High Protein Low Protein		Infection # (%) High Protein Low Protein		Mechanical Ventilation High Protein Low Protein
(1) Clifton 1985	Head injured patients Comatose for 24 h; *N* = 20	C.Random: not sure ITT: yes Blinding: no (8)	22% protein, 38% CHO, 41% fat, 1.5 Kcal/mL (Traumacal vs. 14% protein, 50% CHO, 36% fat, 2.0 kcal/mL (Magnacal) Isocaloric, 29 g Nitrogen vs. 17.6 g nitrogen	Three-month 1/10 (10)	Three-month 1/10 (10)	3/10 (30)	2/10 (20)	NR
(2) Rugeles 2013	Medical adult ICU patients *N* = 80	C.Random: yes ITT: no Blinding: double (7)	Hypocaloric hyperproteic (15 kcal/kg, 1.7 g/kg/day) × 7 days vs. standard (25 kcal/kg, 20% calories from protein).	28 day ^1^ 11/40 (28)	28 day ^1^ 12/40 (29)	NR ^1^	NR ^1^	8.5 ± 4.6 days 9.7 ± 4.9 days (40 patients per group)
(3) Doig 2015	Medical ICU adult patients *N* = 474	C.Random: yes ITT: yes Blinding: no (10)	IV aa infusion (Synthamin, Baxter, 100 g/L) providing a max 100 g aa/day. IV aa infusion was titrated to provide 2 g/kg/day of amino acids from all nutrition sources.	ICU 28/239 (11.7) Hospital 37/239 (15.5) 90-day 42/236 (17.8)	ICU 30/235 (12.8) Hospital 43/235 (18.3) 90-day 47/235 (20)	NR ^2^	NR ^2^	7.33 (7.0–7.68) 7.26 (6.94–7.61) Mean ± SD
(4) Ferrie 2016	Medical/Surgical ICU adult patients *N* = 120	C.Random: yes ITT: yes (modified) Blinding: double (10)	Patients on PN randomized to receive a higher aa vs. lower aa solution with a goal of 1.2 vs. 0.8 g/kg/day aa from EN and PN. Intervention group actually received 1.1 vs. 0.9 g/kg/day in the control group.	ICU 8/59 (14) Hospital 12/59 (20) 6 month 15/59 (25)	ICU 6/60 (10) Hospital 9/60 (15) 6 month 9/60 (15)	31/59 (53)	34/60 (57)	2.0 (1.0–3.0) 2.0 (1.0–5.0) 3.68 ± 6.17 ^3^ 5.87 ± 14.27 ^3^
(5) Allingstrup 2017	Medical/Surgical, Mechanically ventilated ICU adult patients *N* = 203	C.Random: yes (1:1) ITT: yes (modified) Blinding: double (10)	Intervention group received 100% of caloric requirements as derived from indirect calorimetry and protein (minimum; 1.5 g/kg/day) vs. fixed caloric target (25 kcal/kg/day) and protein at 1.2 g/kg/day (control)	28 day 20/100 (20) 90 day 30/100 (30) six months 37/100 (37)	28 day 21/99 (21) 90 day 32/99 (32) six months 34/99 (34)	19/100 (19)	12/100 (12)	6 (4–15) vs. 5 (3–10)

^1^ Response from author 15 December 2016: 28 days mortality: Hyperproteic: 28%, Control: 29%. Other mortality by group: not captured. Number of patients who developed infections, by group: not captured. ^2^ Response from author 22 December2016: We had no reason to suspect infections might be affected, so we did not collect information on infectious complications. ^3^ Response from author 03 February 2017: Days mechanically ventilated, low aa: mean 5.87 (SD 14.27), higher aa: mean 3.68 (SD 6.17). NR, not reported; LOS, length of stay; ICU, intensive care unit; ITT, intention to treat; C. random, concealed randomization; QOL, quality of life; u/s, ultrasound; d/c, discharge; SD, standard deviation; kg, kilograms; cm, centimeter. Intravenous (IV): Parenteral Nutrition (PN): Enteral Nutrition(EN); #, number of patients.
